# Effects of Antimicrobial Peptide Revealed by Simulations: Translocation, Pore Formation, Membrane Corrugation and Euler Buckling

**DOI:** 10.3390/ijms14047932

**Published:** 2013-04-11

**Authors:** Licui Chen, Nana Jia, Lianghui Gao, Weihai Fang, Leonardo Golubovic

**Affiliations:** 1 Key Laboratory of Theoretical and Computational Photochemistry, Ministry of Education, College of Chemistry, Beijing Normal University, Beijing 100875, China; E-Mails: chenlicui@163.com (L.C.); jiananaok@yeah.net (N.J.); fangwh@bnu.edu.cn (W.F.); 2 Physics Department, West Virginia University, Morgantown, WV 26506-6315, USA

**Keywords:** antimicrobial peptides, membrane, dissipative particle dynamics

## Abstract

We explore the effects of the peripheral and transmembrane antimicrobial peptides on the lipid bilayer membrane by using the coarse grained Dissipative Particle Dynamics simulations. We study peptide/lipid membrane complexes by considering peptides with various structure, hydrophobicity and peptide/lipid interaction strength. The role of lipid/water interaction is also discussed. We discuss a rich variety of membrane morphological changes induced by peptides, such as pore formation, membrane corrugation and Euler buckling.

## 1. Introduction

Antimicrobial peptides (AmPs) are one kind of membrane-lytic peptides that serve as defense weapons against bacteria [1]. The AmPs are secreted by organisms of plants and animals and have a wide variety in composition and structure [2,3]. These short chain peptides are usually composed of 12–45 amino acid residues that carry positive charges. When binding onto the outermost leaflet of negatively charged bacteria membranes by the aid of hydrophobic and electrostatic interactions, they will fold to amphipathic secondary structure, such as *α*-helices and *β*-sheets. Their specific structure, amphiphilicity and charge allow them to disrupt the cell functions via either physical, chemical or biological processes. Though peptide killing mechanisms are proposed by several models [4–6], the detailed structure change and corresponding function loss of the bacterial cell are still elusive.

The most commonly accepted picture of the killing process is a physical hole model proposed by Shai, Matsuzaki and Huang (SMH) [4–6]. At a low peptide/lipid (P/L) molar ratio, the peptide binding does not significantly affect the membrane. As the P/L molar ratio increases, the peptides begin to insert into the membrane core in order to release the tension induced by the binding. Thus, hydrophilic pores may form in the membrane and result in membrane permeability. The peptides can translocate across the membrane via the pores and accumulate in the interior of the cell. Due to experimental resolution limits, the actual structure of the pores has not been visualized yet. A few models are introduced to explain the effects of different AmPs: (a) “Barrel-stave model”. In this model, the peptides form a bundle in the membrane with a hydrophilic channel in the center. It was used to explain the unique behavior of alamethicin [6]. The same model was used to elucidate other peptides, for example, trichogin peptide [7]; (b) “Toroidal-pore model”. In this model, the inserted peptides associate with lipid heads to form a pore. In contrast to the barrel-stave model, here, the line of the water pore is composed of both peptides and lipid head groups. Magainins, protegrins and melittins are believed to employ such mechanisms [8]; (c) “Carpet model”. In this model, the peptides spread on the surface of the membrane and cover the surface like a carpet. At high peptide concentration, some peptides insert and translocate across the membrane. The transmembrane and translocated peptides completely surround a part of the membrane and induce the formation of a micelle (in a detergent-like fashion). This model is used to explain the activity of ovisprin [9].

To further understand the mechanisms of AmPs and visualize the intermediate structure, modeling of molecular dynamics is employed to investigate the interactions of peptides and membranes. Such studies may help in developing new peptide-based drugs [10]. In our recent studies [11,12], we applied the Dissipative Particle Dynamics (DPD) with the implementation of electrostatic interaction to the peptide-membrane systems. We found that electric conditions, peptide concentration and peptide/lipid attraction all play important roles in lipid membrane/peptide complexes. In this article, we further systematically investigate the mechanisms and kinetics of the antimicrobial peptide activity by considering peptides with various structure, hydrophobicity and peptide/lipid (P/L) interaction strength. We construct three different peptide models, sorted as peripheral and transmembrane peptides. Peripheral peptides facilitate binding to a single membrane leaflet, whereas transmembrane peptides facilitate leaflet-to-leaflet transmembrane configurations. We also explore the role of water-lipid head interaction. Depending on the strength of this interaction, the lipids are sorted into two classes: “dry lipids” (D-lipids) and “wet lipids” (W-lipids). Peptides affect these two kinds of lipids in adverse fashions described in detail in this paper. For both types of lipids, peptides can induce significant morphological membrane changes in the form of membrane corrugation and Euler buckling. Such membrane states have been indeed seen in a number of experiments [13,14], yet this is the first theoretical study addressing these phenomena more deeply. A corrugated (or buckled) membrane-peptide complex is in an incubation phase preceding the execution phase of antimicrobial peptides action *in vivo*.

## 2. Coarse-Grained Molecule Models

In DPD simulations [15,16], the unit particle or bead is a small fluid volume containing several atoms or molecules. All the beads interact via short-ranged repulsive force, $F_{ij}^{C}=a_{ij}(1-r_{ij}/r_{0}){\hat{r}}_{ij}$, random force, $F_{ij}^{R}=\sqrt{2\gamma_{ij}k_{B}T}(1-r_{ij}/r_{0})\zeta_{ij}{\hat{r}}_{ij}$ and dissipative force, $F_{ij}^{D}=-\gamma_{ij}{\left(1-r_{ij}/r_{0}\right)}^{2}({\hat{r}}_{ij}\cdot v_{ij}){\hat{r}}_{ij}$ for two beads with separation distance, *r_ij_< r*_0_, [16]. Here, the vectors, **v***_ij_≡***v***_i_* − **v***_j_*, are the velocity differences between particles, *i* and *j*. The parameters, *a_ij_*, represent the repulsion strengths. γ*_ij_* are the friction coefficients. *ζ_ij_* are symmetrically and uniformly distributed random numbers. In addition, for polymeric molecules, such as lipids and peptides, beads are connected by harmonic Hookean spring forces, and bending stiffness forces are also included [11,12]. Electrostatic interactions between charged beads are calculated by using the method introduced by Groot [17] and recently further developed by us [18], where the charges are distributed on a lattice and the electrostatic field is solved locally on the grid. Some of the here reported simulations are performed in constant particle number, surface tension, normal pressure and temperature (*Nγ_s_P*_⊥_*T*), assembled by using the Langevin piston method to control the pressure [19]. We also describe the results obtained with constant simulation box sizes, *i.e*., the (*NV T*) ensemble. In addition, we report our results obtained by simulating lipid membrane vesicles interacting with peptide molecules. The detailed descriptions of the simulation approach can be found in our recent papers [11,12].

**Figure 1:**
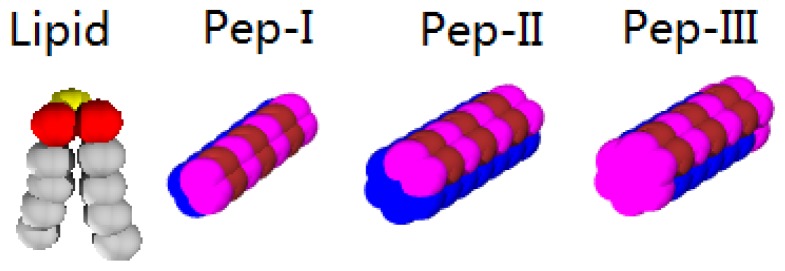
Models of lipid and antimicrobial peptide. Lipid molecules have two hydrophobic chains (gray) and three head beads (neutral beads are red, and negatively charged beads are yellow). Pep-I is a cylindrical bundle composed of two hydrophilic and two hydrophobic chains; Pep-II is a bundle composed of two hydrophilic and five hydrophobic chains; the bundle of Pep-III is the same as the bundle of Pep-II, but the end caps are all set to be hydrophilic. The hydrophobic part of the peptide is in blue, and the hydrophilic part is in purple and brown. Each brown bead carries a +0.5e charge.

The system we are studying includes lipid bilayer membrane, peptides, explicit water and the counterions of lipids and peptides. In the DPD simulations, water (denoted by *W*) is modeled as a single bead corresponding to three water molecules. The lipid molecule is coarse-grained as a polymer with three hydrophilic head beads (denoted by *H*) and two hydrophobic carbon chains (denoted by *C*), as shown in Figure 1. For a negatively charged lipid, one of the head bead carries a net charge, -e. In Figure 1, we also present three kinds of coarse-grained *α*-helix peptides. Pep-I represents a relatively flexible peptide, which is modeled as a bundle of four chains interacting through harmonic forces. Each peptide chain has seven beads. Two adjacent chains are hydrophilic (denoted by *Ph*) and positively charged, with a net charge of +3e; the other two chains are hydrophobic (denoted by *Pc*). Pep-II is a more rigid peptide modeled as a cylinder composed of seven chains. Five of the chains are hydrophobic. It thus exhibits a significant hydrophilic/hydrophobic asymmetry. Pep-III is similar to Pep-II, but with the end beads set to be hydrophilic. Peptides with this structure naturally facilitate a transmembrane state. Lipid and peptide counterions are both single beads carrying a positive, +e, or a negative, -e, charge, respectively.

The force parameters, *a_ij_*, governing the repulsion between the beads are given in Table 1. Other parameters can be found in Refs. [11,12]. In the DPD simulations, the force parameters are obtained by fitting to the physical properties of the system, such as the water pressure and lipid structure and diffusivity [20]. In this article, we mainly modify the repulsion strength, *a_PhC_*, between the hydrophilic bead of peptide and the hydrophobic bead of lipid carbon chains to describe the P/L interaction. We choose *a_PhC_* = 25, 35, 50 (in the units of *k_B_T=r*_0_) to represent weak, intermediate and strong P/L repulsion. We also tune the parameter, *a_PcW_*, from 120 to 500 to represent weak and strong hydrophobicity of the peptide. In the following sections, we describe the results obtained with two different types of lipids, sorted according to the strength of their repulsive interaction with water. We label them as “dry lipids” (D-lipids) and “wet lipids” (W-lipids). For D-lipids, *a_HW_* = 35 *> a_HH_* = *a_WW_* = 25. For W-lipids, *a_HW_* = 20 *< a_HH_* = *a_WW_* = 25. As detailed in this paper, there are substantial differences between D-lipid/peptide complexes and W-lipid/peptide complexes.

Initially, a planar bilayer membrane composed of 1,600 lipids is placed in the center of a simulation box with the size 32*r*_0_*×* 32*r*_0_*×* 32*r*_0_ (*r*_0_ = 0:76 nm). Thirty percent of the lipids are negatively charged and distributed randomly on both leaflets of the membrane to mimic the bacterial membrane. Four-hundred eighty lipid counter ions and 80,224 water beads are distributed randomly in the space unoccupied by the membrane. The system is relaxed for 50,000 time steps (1 *μ*s) at zero surface tension to reach equilibrium. Then, eight peptides and their counter ions are added into the system. They are placed about 2 nm away from the lower (in our figures) leaflet of the membrane. (In the text, the lower leaflet is referred to as the outer leaflet; the upper leaflet is referred to as the inner leaflet.) At the same time, some water beads are removed from the system, so that the total number of all beads remains the same. This peptide-membrane system is relaxed for 1 *μ*s. Then another eight peptides are added into the system and simulation is continued for another 1 *μ*s. This procedure is repeated four times till the system has 32 peptides, *i.e*., the P/L molar ratio reaches 2/100. After this, we perform long simulations (up to 7 *μ*s) to investigate the kinetics and dynamics of the peptide-membrane system. Because the coarse-grained peptides are in a secondary structure even in water, they are added into the simulation box step by step in order to avoid strong peptide association before they bind onto the membrane. For each of the three kinds of peptide (Pep-I, Pep-II, Pep-III), at least five independent simulations are performed to collect data.

## 3. D-Lipid/Peptide Complexes

In this section, we will describe the behavior of D-lipid membranes in the presence of the three kinds of peptides introduced in the previous section. Unless specified otherwise, the results in this section are displayed from our simulations in the (*Nγ_s_P*_⊥_*T*) ensemble at zero global tension (see, however, the end of Section 3.1). The membrane rigidity constant is about 12*k_B_T*. Isotropic pressure of $23k_{B}T/r_{0}^{3}$ is applied (it corresponds to having about three real water molecules per single water bead volume).

### 3.1. Flexible Peptides with Hydrophilic/Hydrophobic Symmetry (Pep-I Model)

**Figure 2:**
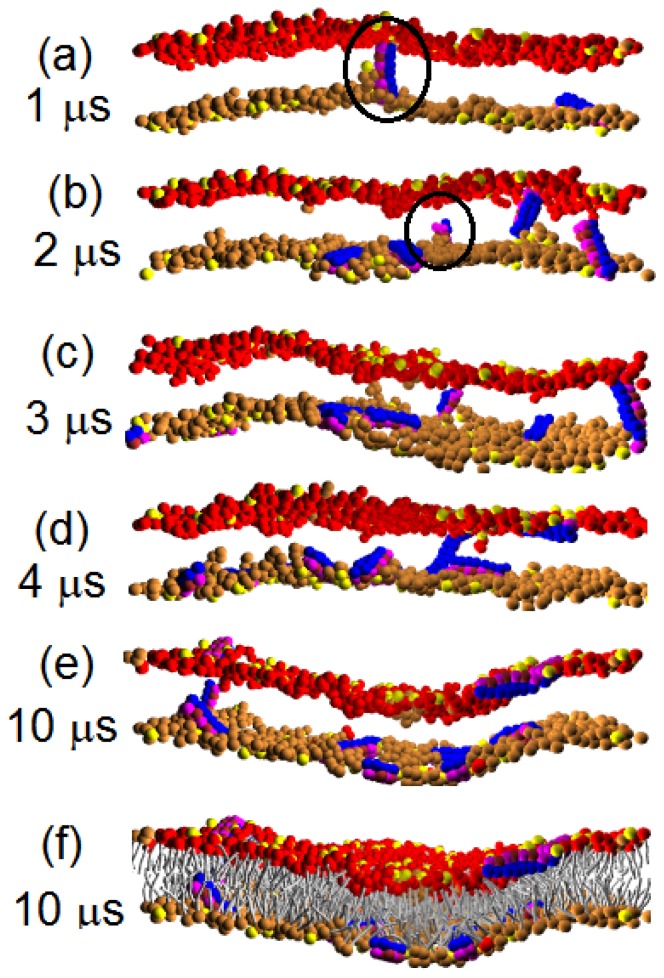
(**a**)–(**e**) Time sequence of the cross sectional image for Pep-I interacting with the dry lipid (D-lipid) bilayer membrane simulated with force parameter, *a_PhC_* = 35. For clarity, lipid tails and water are not shown; (**f**) The complete view of the whole peptide-bilayer complex with lipid tails shown. Note: The neutral head beads in the lower monolayer are in the color gold to distinguish the upper monolayer.

Pep-I is a bundle composed of only four chains; see Figure 1. It is thin and more flexible in comparison with Pep-II and Pep-III. The time sequences of the snapshot of the peptide/membrane complex simulated with force parameter, *a_PhC_* = 35, are given in Figure 2. (For a clear view, the membrane is cut into four slices. The snapshots are the cross-sectional images of one of the slices.) One can see notable differences between the early times when the P/L ratio is less than 2/100 and the late times when the P/L ratio is kept at 2/100. Already at the early times, the peptides bind onto the surface of the membrane and disorder the lipids around them. Such binding induces local compression on the peptide-rich leaflet of the membrane and a tension on the peptide-poor leaflet [18]. Some peptides then tend to intrude into the membrane interior to release the local tension; see the circled region in Figure 2a. However, at early times (*i.e.*, at low P/L ratios), these peptides are pulled back to the membrane surface again, as seen in Figure 2b. This is because at a low P/L ratio, the local tensions (compressions) can be relaxed to a weaker global tension (compression) via lipid diffusion. The difference between the global tension of the inner leaflet and the global compression of the outer leaflet may not be high enough to induce peptide insertion into the membrane. So, at low P/L ratios, the peptides mostly remain in the parallel (to membrane) adsorption state. However, once the P/L ratio reaches 2/100, the difference between the stress states of the leaflets becomes strong enough to induce the insertion of peptide molecules into the membrane interior. This insertion state is, however, only metastable, because the electrostatic attraction between the peptide and the acidic lipid heads on the inner membrane leaflet further promotes the peptide to move toward the other side of the bilayer and reach to the final parallel adsorption state; see Figure 2d. In this way, a peptide molecule is capable of translocating across the membrane via a transient wormhole. At each time, only one peptide is found in a transient pore, as shown in Figure 2. In the five samples simulated up to 10 *μ*s, up to 10 peptide translocations are observed, see Table 2.

**Figure 3:**
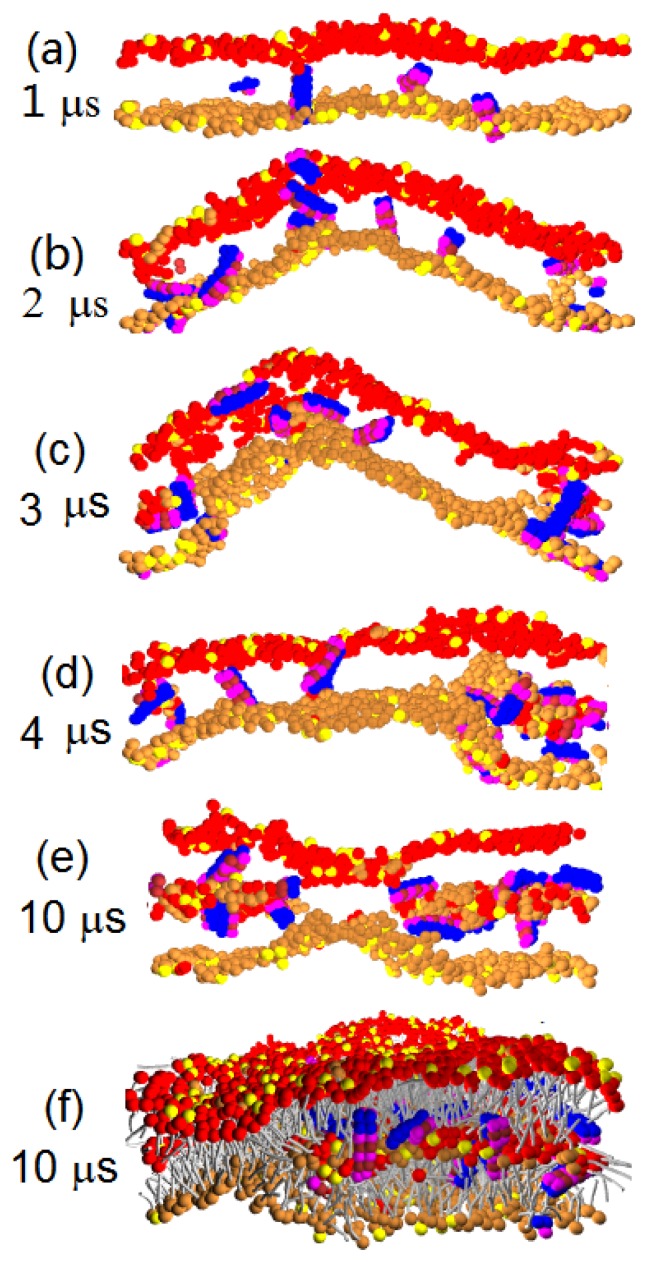
(**a**)–(**e**)Time sequence of the cross sectional image for Pep-I interacting with the D-lipid bilayer membrane simulated at *a_PhC_* = 25. Lipid tails are shown only in (**f**).

Next, we perform simulations with a lower P/L repulsion parameter, *a_PhC_* = 25. With this parameter value, the peptides have a strong affinity to insert into the membrane core and stay deeply inside, because the P/L repulsion is weaker than the repulsion between water and hydrophilic peptide beads (*a_PhC_< a_PhW_* = 35); see Figure 3a,b. Remarkably, the intruding peptides can also attach lipid heads to enter into the membrane (see Figure 3c,d) and form a new layer inside the membrane, as shown in Figure 3e,f. The new layer has a hydrophilic core (comprised of lipid heads associated with peptides) and a hydrophobic hair (comprised of lipid tails). The hydrophilic core is much like the inner shell of a discoidal vesicle. Here, in contrast to an ordinary discoidal vesicle, the outer shell of the vesicle-like structure is not closed; see Figure 3e,f. Rather, it is continuously connected to the leaflets of the bilayer membrane. The hydrophilic core occasionally comes in contact with membrane leaflets to accumulate more peptides and lipids. So, after its nucleation, the discoidal hydrophilic core continues to grow. We find that the growth of the discoidal core actually inhibits the peptide translocation. In five independent samples simulated up to 10 *μ*s, there are only 5–6 peptides on average observed to translocate (see the statistics in Table 2). In one sample, even no peptide translocation is observed. Most of the peptides actually become trapped inside the discoidal core. Only a few peptides remain on the outer (lower) leaflet of the membrane, as seen in the snapshots from our simulation. Importantly, as shown in Figure 3, the presence of the discoidal core causes a substantial corrugation of the multilayered membrane structure. In Figure 3 at 3 *μ*s, we see a very strong membrane undulation. This strong undulation is, however, only a transient effect. At later times, the magnitude of the undulation decreases as the core grows. In effect, at later times, the magnitude of the membrane corrugation is basically the same at the thickness of the new lipid layer growing inside the membrane.

**Figure 4:**
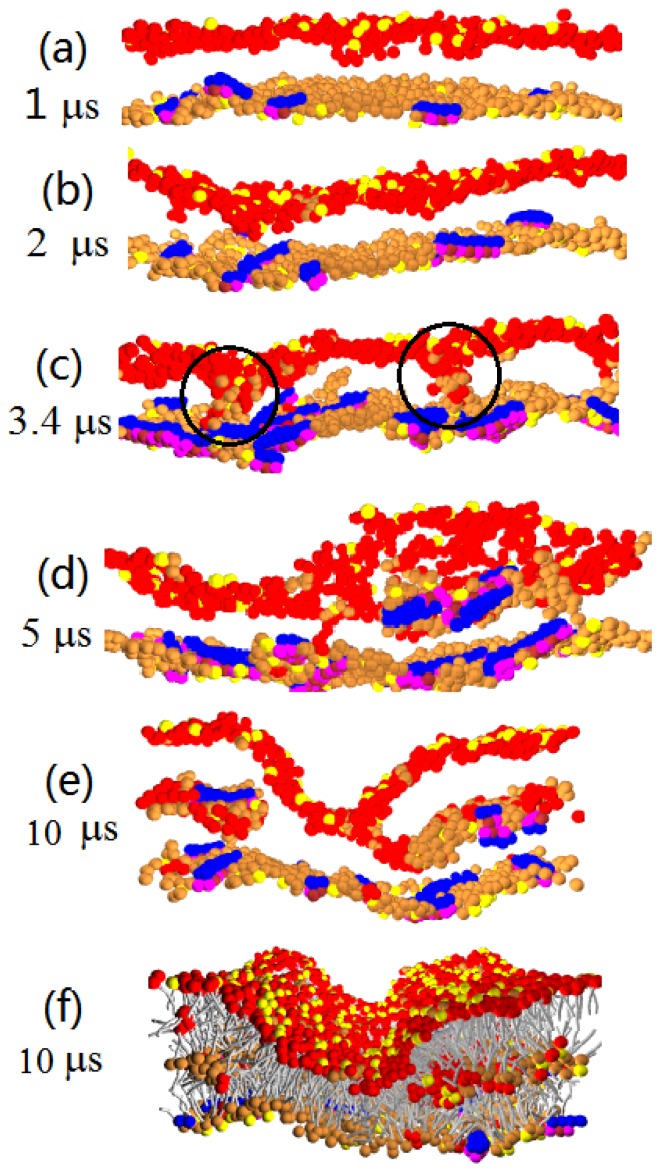
(**a**)–(**e**) Time sequence of the cross sectional image for Pep-I interacting with the D-lipid bilayer membrane simulated at *a_PhC_* = 50. Lipid tails are shown only in (**f**).

We also perform simulations with a higher P/L repulsion parameter, *a_PhC_* = 50. The snapshots are given in Figure 4. In this case, the peptide has low affinity to insert into the hydrophobic core of the membrane, and a few or even no peptide translocation is observed; see Table 2. To relieve the compressional stress on the peptide-rich leaflet of the membrane, lipid heads are squeezed into the hydrophobic interior of the bilayer and initially form pore-like objects; see Figure 4c. Their size, however, continues to increase by absorbing additional lipids and peptides. Thus, much like at *a_PhC_* = 25, at *a_PhC_* = 50, we also find the development of hydrophilic cores and multilayered corrugated structure. At *a_PhC_* = 25, however, the core is mainly composed of peptides, whereas at *a_PhC_* = 50, the lipid heads are the dominant core components, while most of the peptides remain on the outer peptide-rich leaflet of the membrane.

**Figure 5:**
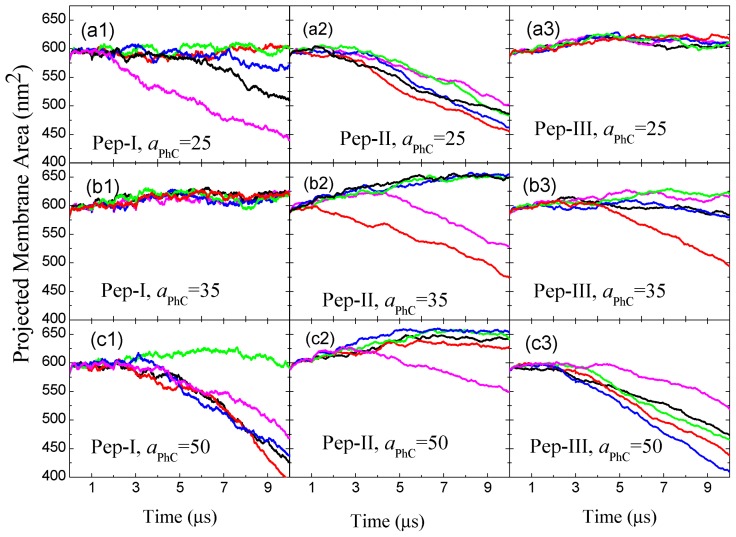
Time evolution of the projected membrane area induced by the binding of Pep-I, Pep-II and Pep-III, each simulated at *a_PhC_* = 25, *a_PhC_* = 35 and *a_PhC_* = 50. Five independent samples are given for each of the nine cases.

**Figure 6:**
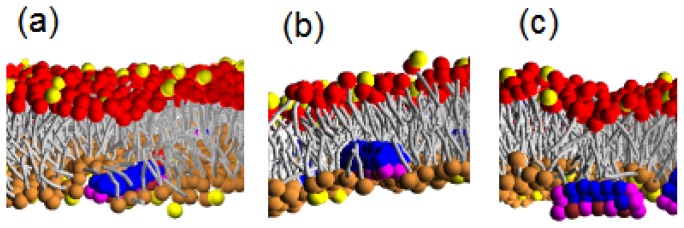
The penetration depth of (**a**) Pep-I; (**b**) Pep-II and (**c**) Pep-III into the D-lipid bilayer in the binding state simulated at *a_PhC_* = 50.

The hydrophilic cores have significant effects on the projected area of the membrane (or the simulation box size parallel to the initial membrane configuration); see Figure 5. Over the early stage of the simulation, the membrane area increases due to peptide adsorption. The membrane area increase is not large, since the peptides only deform the lipids underneath them by changing the tail’s orientation from normal to parallel to the membrane surface; see also Figure 6a. The membrane is still nearly flat at early times. However, once the discoidal hydrophilic core nucleates (in three out of five samples at *a_PhC_* = 25, and in four out of five samples at *a_PhC_* = 50), it accumulates lipids and peptides from both leaflets of the membrane and continues to grow in size. This growth produces a steady decrease of the projected area of the membrane seen in Figure 5a1,c1 at *a_PhC_* = 25 and at *a_PhC_* = 50, respectively. For the Pep-I systems with *a_PhC_* = 35, the plots of the projected area in Figure 5b1 indicate that there is no hydrophilic core nucleated inside the membrane (in all five simulated samples). For this case, translocation of the peptide is the main pathway for the membrane to release peptide induced local stresses. This is consistent with the statistics of the hydrophilic core formation and translocations rates in Table 2, given for the three studied values of *a_PhC_*. By the table, we can see that core formation and translocation appear as two competing kinetic processes. At *a_PhC_* = 35, translocation wins, whereas at *a_PhC_* = 25 and at *a_PhC_* = 50, core formation wins.

**Figure 7:**
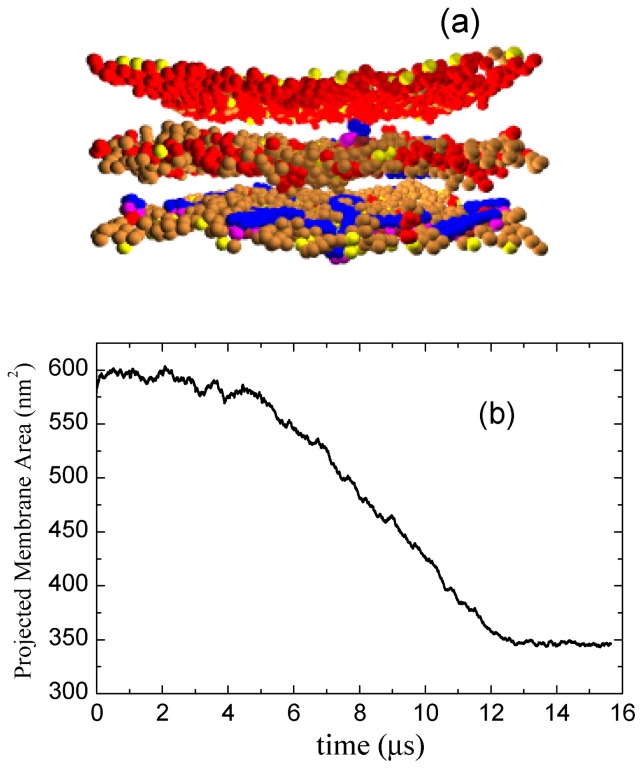
(**a**) The snapshots of a well-ordered multilayered D-lipid membrane with 400 lipids induced by the binding of Pep-I simulated with *a_PhC_* = 50 at 20 *μs*; (**b**) Time evolution of the projected D-lipid membrane area.

The discoidal hydrophilic cores nucleated inside membranes eventually develop into full layers and result in a multilayered membrane structure seen in our zero surface tension simulations at the longest times. Figure 7a gives an example of this multilayered structure at the simulation time of 16 *μ*s at *a_PhC_* = 50. The plot of the projected membrane area as a function of time, Figure 7b shows that the area approaches equilibrium at 12 *μ*s when a well ordered multilayer structure is formed in this particular simulation. (In other four simulations, it took more time for full layers to develop.) The final projected (base) area is about 60% of the initial area. From our simulations with different membrane sizes (not shown), we find that the full equilibration time increases with increasing membrane size. We recall that these results are obtained from the simulations done in the (*Nγ_s_P*_⊥_*T*) ensemble at zero global tension. We also did the simulations of the same system in the (*NV T*) ensemble, *i.e*., with constant simulation box sizes. In this ensemble, the membrane base area is held constant. We find that the cores form also in this ensemble. However, the growth of a core is eventually halted by a tension the core induces in the rest of the membrane (by sucking lipids out of it). In effect, the final core size never reaches the full membrane size. We also did a set of simulations with D-lipid vesicles. Cores nucleated in these vesicles induce a significant nonsphericity, as well as a membrane tension. The resulting Laplace pressure causes a substantial transmembrane water flux out of the vesicles. We will describe the results with D-lipid vesicles in more detail elsewhere.

### 3.2. Rigid Peptides with Hydrophilic/Hydrophobic Asymmetry (Pep-II Model)

Another property that may potentially affect the mechanism of activity of the peptides is their hydrophobicity. Yet, by changing the corresponding force parameter, *a_PcW_*, from 120 to 200 and 500 for Pep-I, we did not observe any qualitatively significant effects. Then, we designed the model of Pep-II with hydrophilic/hydrophobic asymmetry, see Figure 1. Note that the hydrophobic part of Pep-II is bigger than that of Pep-I. The big hydrophobic portion of the Pep-II also makes it repel the lipid head more strongly (here *a_PcH_* = 50). As discussed below, Pep-II molecules exhibit diverse activities with changing P/L repulsion strength.

**Figure 8:**
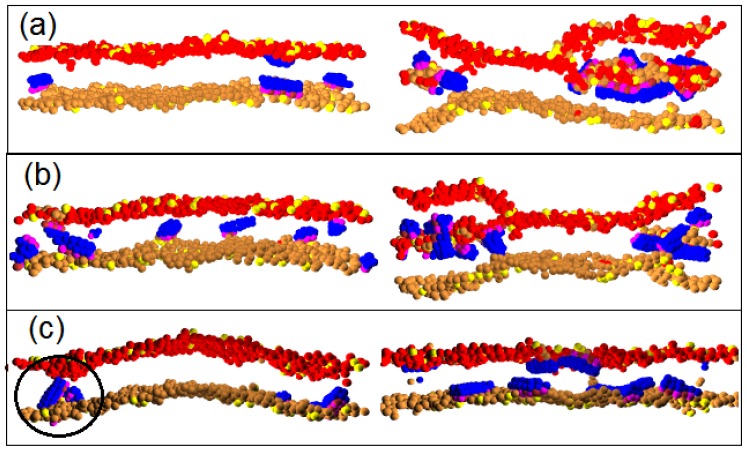
The cross sectional images for Pep-II interacting with the D-lipid bilayer membrane simulated at (**a**) *a_PhC_* = 25; (**b**) *a_PhC_* = 35 and (**c**) *a_PhC_* = 50. In the three panels on the left, the snapshots are at 1 *μs*; in the three panels on the right, the snapshots are at 10 *μs*.

The high hydrophobicity and larger diameter of Pep-II permit them to penetrate deeply into the hydrophobic membrane interior by taking up the positions of lipids underneath them and strongly compressing the surrounding lipids, as can be seen in Figure 6b. Thus, a Pep-II molecule may *a priori* have better chances to move across the membrane, in comparison to Pep-I. Indeed, we could see Pep-II translocation even at a small P/L molar ratio, such as 1/100; see the left panel of Figure 8a,b. Nonetheless, somewhat strikingly, with increasing P/L molar ratio (to 2/100), less translocations are actually observed for the systems with *a_PhC_* = 25 and *a_PhC_* = 35; see Table 2. The strong hydrophobicity of Pep-II prevents them from reaching to the inner surface of the membrane. Rather, they prefer to associate with each other, as well as with lipids to form a new layer inside the membrane; see the right panel of Figure 8a,b. These morphology changes are reflected in the plot of the projected membrane area as a function of time in Figure 5. For the Pep-II systems with *a_PhC_* = 25, the decreasing of the projected membrane area shows that a hydrophilic core with peptide-lipid association forms in all of the five samples; see Figure 5a2. For the Pep-II systems with *a_PhC_* = 35, the core structure are observed in two out of the five simulated samples, Figure 5b2.

In contrast to Pep-I, at high P/L repulsion with *a_PhC_* = 50, we did observe more translocation events for Pep-II (see Table 2). A high P/L repulsion and the deep penetration of larger size Pep-II molecules conspire to inhibit the concurrent entrance of lipids into the membrane interior (hydrophilic core formation) seen with smaller sized Pep-I molecules. Thus, to release the strong compression of the membrane induced by the binding of the large size Pep-II molecules, more peptides are essentially required to move alone (without accompanying lipids) across the membrane. Hence, typically, no hydrophilic core is formed in this case. Indeed, from the plots of the membrane area given in Figure 5c2, we can see that core structure emerges in only one out of five samples. Thus, at a high P/L repulsion, the hydrophilic core nucleation is a rare event for Pep-II, in marked contrast to Pep-I behavior at high P/L repulsion; see Figure 5c1. An important feature we would like to emphasize here is that Pep-II molecules tend to associate when they insert into the membrane at a high P/L repulsion; see Figure 8c at 1 *μs*. This association prevents the hydrophilic faces of the peptide to come in contact with the hydrophobic lipid tails to minimize the energy-cost of the insertion. The cooperative peptide association strongly deforms the order of the surrounding lipids and promotes more peptide translocation events.

### 3.3. Transmembrane Peptides (Pep-III Model)

**Figure 9:**
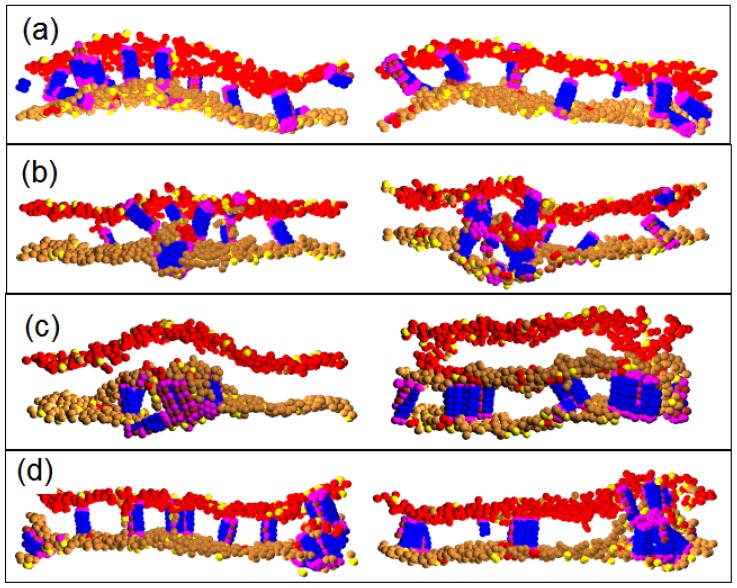
The cross sectional images for Pep-III interacting with the D-lipid bilayer membrane simulated at (**a**) *a_PhC_* = 25; (**b**) *a_PhC_* = 35 and (**c**) *a_PhC_* = 50; (**d**) gives the snapshots for peptides having only one hydrophilic chain with *a_PhC_* = 50. The four panels on the left give the snapshots at 5 *μs*; the four panels on the right give the snapshots at 10 *μs*.

Pep-III is a rigid peptide with hydrophilic caps at the two ends of the cylinder; see Figure 1. The caps lower the hydrophobicity of the peptide so that the binding penetration is shallow, as shown in Figure 6c. Yet, due to the hydrophilic caps, once it manages to insert, a Pep-III molecule will strongly tend to remain in the transmembrane insertion state bridging across the membrane; see Figure 9. This transmembrane affinity inhibits the peptide translocation. The statistics of translocation in Table 2 supports this conclusion.

Let us discuss the evolution of the peptide-membrane complex for the Pep-III model. For systems with *a_PhC_* = 25, the weak P/L repulsion permits the peptides to insert into the membrane and form stable transmembrane wormholes; see Figure 9a. There, we also see that the presence of inserted peptides causes enhanced membrane undulations. The peptide insertion somewhat increases the membrane area, as seen in Figure 5a3. At *a_PhC_* = 35, stable transmembrane wormholes are formed too; see Figure 9b. In this figure, we see another important peptide effect: we find that that the local stresses (induced by the adsorbed peptides) promote some lipid heads to invade the membrane interior. There, they nucleate a hydrophilic core, and a new layer starts to grow. The trapped layer further attracts the transmembrane peptides to gather around it; see Figure 9b. Finally, a complex structure with three lipid head layers connected by peptide linkers is formed. In contrast to the multilayered corrugated structure induced by Pep-I and Pep-II where the trapped layer is composed of both lipid heads and peptides, in Pep-III systems, the peptides are absent in the hydrophilic core. The core is comprised purely of lipid heads, whereas the peptides serve only to link the core to the upper and lower monolayer, as seen in Figure 9b. The nucleation rate of this core increases with increasing *a_PhC_*. From the plots of the projected membrane in Figure 5b3, we can see that the core was nucleated in one of the five samples at *a_PhC_* = 35, whereas (within our simulation time limits) no core was nucleated in the five samples at *a_PhC_* = 25; see Figure 5a3. In comparison, at *a_PhC_* = 50, the hydrophilic cores are nucleated in all five samples; see Figure 5c3.

We proceed to discuss in more detail our simulations in Figure 9c at *a_PhC_* = 50. The combined effects of the strong P/L repulsion and relatively weak peptide hydrophobicity conspire to push the light weighted lipid head groups to vigorously enter into the membrane interior. A new hydrophilic layer quickly forms inside the membrane interior so that the projected membrane area starts to rapidly decrease, as shown in Figure 5c3. Later on, the transmembrane tendency of the peptides permits them to follow the insertion of lipid and enter into the membrane, too. But in this case, most of the peptides only link the outer monolayer to the center layer, and a few peptides link the inner monolayer to the center layer. We find that the strong P/L repulsion also promotes the peptides to associate into bundles with their hydrophilic faces in the bundle center. In the early stage, these bundles resemble the toroidal pores composed of both peptides and lipids. However, when the newly formed hydrophilic layer becomes saturated with lipids, the morphology of the peptide bundles becomes similar to that of the barrel-stave model. Such barrel-stave bundles composed of 4–5 peptides are commonly observed in our simulations of Pep-III at *a_PhC_* = 50; see the right panel of Figure 9c. Interestingly, more than 20 water beads are found in the newly formed center layer. This water is likely brought from outside by attaching to the hydrophilic end caps of Pep-III. We cannot, however, rule out the possibility that some of the water observed in the center layer came through the centers of the barrel-stave peptide bundles, which would indicate that the peptide bundles connecting the outer leaflet and the center layer are water permeable. However, close inspection of the peptide bundles shows that they do not have well-formed water channels at their centers. Thus, the water permeability of the barrel-stave peptide bundles is small.

The above described peptide bundles may provide only an indirect connection (across the center layer) of the cell with its exterior. The observation of these indirect barrel-stave bundles stimulates an interesting question: is there any kind of peptide such that it can induce the standard barrel-stave peptide bundles directly bridging between the inner and outer monolayer of a D-lipid bilayer? For this purpose, we consider a model peptide similar to Pep-III in Figure 1, but with only one hydrophilic chain in its bundle. Thus, the mid part of the molecule becomes more hydrophobic. In this model simulated at *a_PhC_* = 50, both direct and indirect barrel-stave peptide bundles are observed in a single sample; see Figure 9d. Comparing this figure with Figure 9c, we also see that the increase of the hydrophobicity has the effect to depress the rate of the hydrophilic core nucleation. Finally, we note that direct barrel-stave peptide bundles (much like the indirect ones) do not have well-formed water channels. Thus, the water permeability of both direct and indirect barrel-stave peptide bundles is small.

## 4. Discussion of D-Lipid/Peptide Complexes

From the results in the previous sections, we see that different peptides with various peptide/lipid repulsion strength, structure and hydrophobicity exhibit different mechanisms capable of affecting D-lipid membrane structure and functions. In this section, we discuss these peptide physical effects on D-lipid bilayer membranes.

### 4.1. Peptide/Lipid Repulsion

The peptide/lipid interaction is mainly determined by the force parameter, *a_PhC_*. The weak repulsion (low *a_PhC_*) means that the hydrophilic part of the peptide can penetrate into the hydrophobic membrane interior easily. In the weak P/L repulsion systems, all of the three kinds of peptide we designed here can insert into the membrane individually via a wormhole pore if the P/L molar ratio exceeds a critical value. The inserted peptides either stay inside the membrane core by associating with lipid heads or translocate across the membrane to release the tension.

On the other side, at a high value of *a_PhC_*, it costs more free energy for the peptide to insert into the hydrophobic membrane interior than to stay on the membrane surface. So, the binding state is more stable than the insertion state. Indeed, by comparing the last snapshots in Figures 2, 3, 4, as well as those in the right panel of Figure 8, we can see that most of the peptides remain in the inactive absorption state at *a_PhC_* = 50. On the other hand, no peptides remain in the absorption state at *a_PhC_* = 25.

We note that, much like the interaction, *a_PhC_*, the interaction between the hydrophilic peptide portion and lipid heads, *a_PhH_*, also significantly affects the peptide/lipid complexation. Reducing the value of *a_PhH_* has similar effects as increasing *a_PhC_*. For example, with *a_PhC_* = 35 and *a_PhH_* = 10, discoidal hydrophilic cores nucleate faster than in the simulations with *a_PhH_* = 25. A detailed discussion of this effect can be found in our work, Reference [12].

### 4.2. Peptide Hydrophobicity

The hydrophobicity of the peptide determines their penetration depth in the insertion state. Comparing the snapshots for the three types of peptides in Figure 6 (all simulated at *a_PhC_* = 50), we can clearly see that Pep-II molecule with a larger hydrophobic portion can deeply penetrate into the membrane. Pep-II molecule effectively pushes away the lipids and occupies their positions. Its hydrophobic face can even touch the tails of lipids in the distal (inner) monolayer. In Pep-II presence, the bilayers become thinned and highly stretched, as evidenced by the projected membrane area evolution in Figure 5b2 at *a_PhC_* = 35 and c2 at *a_PhC_* = 50. (In the later case, the stretching was suppressed in one of the five samples, because of hydrophilic core nucleation in this sample.) At low P/L molar ratios, the Pep-II molecules can switch from a binding state to an insertion or translocation state. At higher P/L ratios, the Pep-II molecules tend to associate with each other and remain in the membrane interior. Overall, these results indicate that peptides with a larger hydrophobic portion efficiently perturb the local order and function of the membrane.

We stress, however, that in terms of more global morphological effects, such as the nucleation of new hydrophilic membrane layers, the peptide hydrophilicity actually plays a more significant role. Recall our results for the Pep-III model with two hydrophilic chains, which is a more efficient hydrophilic core former than the modified Pep-III with one hydrophilic chain; see Figure 9c,d. The likelihood of hydrophilic core formation increases with increasing peptide hydrophilicity. Likewise, increasing hydrophobicity of peptide decreases the likelihood of hydrophilic core formation. This rule of thumb, however, generally applies only at strong enough P/L repulsion (in our systems with *a_phc_* = 50). For a weak P/L repulsion system, however, increasing peptide hydrophobicity frequently helps the hydrophilic core to be formed. For example, no core formation was observed in the less hydrophobic Pep-I at *a_phc_* = 35, whereas in the more hydrophobic Pep-II at *a_phc_* = 35, core formation was seen in two out of the five simulated samples; see Table 2.

### 4.3. Peptide Structure

The three peptide models designed here mimic two types of secondary structure of peptide: Pep-I and Pep-II are peripheral type peptides having well separated hydrophilic and hydrophobic segments. They facilitate binding states on the lipid head surface with their orientation perpendicular to the bilayer normal. Pep-III is a transmembrane type peptide that facilitates the insertion state in parallel to the bilayer normal. We find that peripheral and transmembrane peptides exhibit notable differences in their modes of action. At high peptide concentration, the peripheral peptide can affect membranes by either translocating across the membranes or by associating with lipids to form a new hydrophilic layer inside the membranes interior. In both processes, the peripheral peptides insert into membranes via wormholes (at low P/L repulsion) or toroidal-like pores (at high P/L repulsion). The pores formed by peripheral peptides in D-lipid membranes are almost non-permeable to water and metastable with a lifetime scale only in tens of nanoseconds. In contrast to this, the transmembrane peptides disturb membranes by forming stable barrel-stave peptide bundles, seen in Figure 9. They can produce some significant water leakage only if they directly connect the inner and outer membrane leaflet. The formation of such direct barrel stave bundles is favored by increasing transmembrane peptide hydrophobicity. With decreased transmembrane peptide hydrophobicity, the formation of new hydrophilic layers is favored, and most of the barrel-stave peptide bundles are indirect, *i.e*., they are formed between the outer membrane leaflet and the new layer, whereas fewer bundles connect the new layer with the inner membrane leaflet. Such D-lipid membrane/peptide complexes (with indirect barrel-stave bundles) would exhibit highly hindered water leakage and, concurrently, a corrugated membrane morphology induced by the presence of the newly formed growing hydrophilic layers.

### 4.4. Possible Killing Mechanisms of Peptide Complexed with D-Lipids

First, let us look at the peripheral peptide complexes with D-lipid membranes. These complexes exhibit only short-lived membrane pores facilitating peptide translocation. These pores are poorly permeable to water, so the cell death by water leakage is unlikely to occur in D-lipid complexes with peripheral peptide molecules. Thus, the peptide insertion state itself may not correspond to the final, execution stage of microbial death. For the systems in which peptide translocation dominates, a possible killing mechanism is that the translocated peptides accumulate in the interior of the cell and affect metabolic processes therein. Let us next look at transmembrane peptide complexes with D-lipid membranes. These complexes exhibit permanent barrel-stave peptide bundles, which, however, do not have well-formed water channels. Due to small water permeability, these complexes cannot induce a fast cell death by leakage.

However, the D-lipid/peptide complexes developing a corrugated morphology (with an underlying hydrophilic core) may exhibit some alternative killing mechanisms. The core structure is very stable, but still not permeable to water, so the lesion caused by the corrugation of the membrane may not be itself sufficient to lead to microbial death. However, we note that the hydrophilic cores are mainly composed of peptides or lipids from the outer leaflet of the bilayer. Thus, as the hydrophilic cores grow, the normal distribution of the lipids between the two leaflets of the bilayer becomes significantly scrambled. Such scrambling may destroy the trans-membrane potential and cause partial loss of the viability of the cell. In addition, even though no water pore nucleation is observed within our simulation time, lethal cell leakage is still possible to occur: in Figure 3f and Figure 4f, we can see that the membrane is highly curved along the rim of the outer shell of the vesicle-like structure. Pore nucleation and/or fracture of individual leaflets are favored along this rim. Once this happens, a true discoidal vesicle will form. Detachment of this discoidal vesicle will leave behind a large hole in the bilayer. Such a membrane disintegration is somewhat similar to the “carpet model”. However, in contrast to the carpet model, where trans-membrane and translocated peptides completely cover a part of the membrane and induce the formation of a micelle, in our vesicle-like model, the rim of the vesicle does not need to be massively covered by peptides.

## 5. W-Lipid/Peptide Complexes

In this section, we explore the role of water-lipid interaction. As detailed in the following, the main outcome is that (depending on the strength of this interaction) the lipids can be sorted into two classes: “dry lipids” (D-lipids) and “wet lipids” (W-lipids). Peptides affect these two kinds of lipids in adverse fashions. In the previous sections, we have already described the D-lipid/peptide complexes, and now, we turn our attention to the W-lipid/peptide complexes. As discussed in Section 2 (see also Table. 1 caption), W-lipid heads interact with water through a weaker repulsive interaction than D-lipid heads. In view of this, it is to be expected that water molecules better associate with W-lipid heads than with D-lipid heads. As demonstrated hereafter, this feature produces some substantial differences between D-lipid/peptide complexes and W-lipid/peptide complexes. In the following, we report two different sets of simulations we did with W-lipids. In the first set, we simulated W-lipid bilayer membrane/peptide complexes in the (*NV T*) ensemble, *i.e*., with a fixed membrane base area. In the second set of simulations, we simulated interactions between W-lipid vesicles and peptide molecules.

### 5.1. W-Lipid Bilayers Interacting with Peptide Molecules

**Figure 10:**
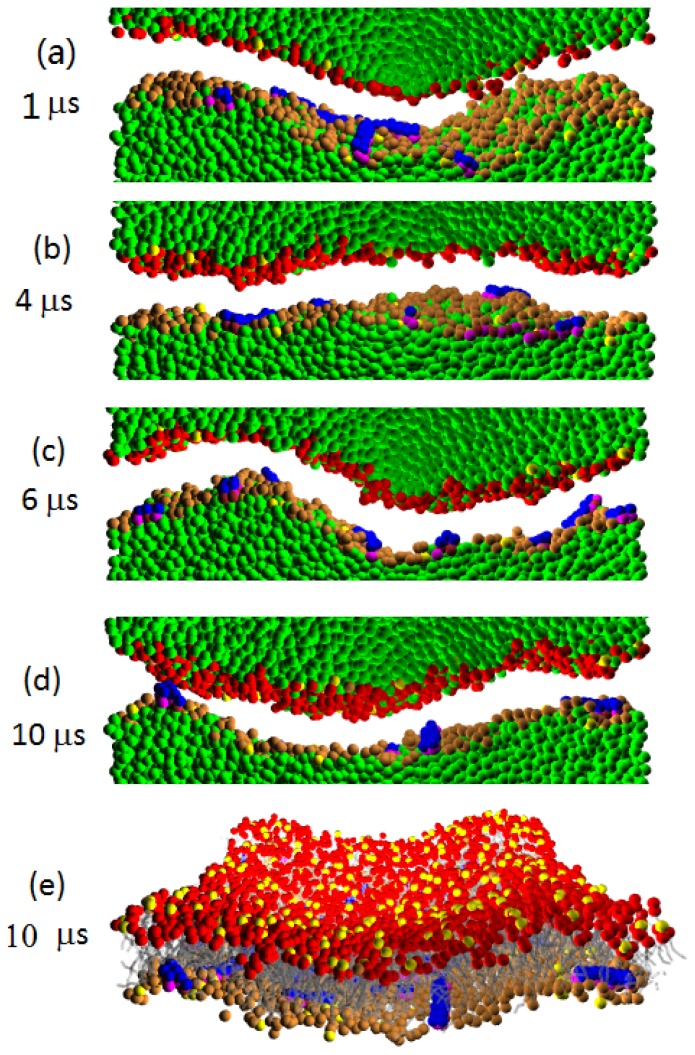
(**a**)–(**d**) Time sequence of the cross sectional images for Pep-I interacting with a W-lipid bilayer membrane simulated at *a_HW_* = 20 and *a_PhC_* = 50. In the cross sectional images, water is explicitly shown, while lipid tails are not shown for clarity. The peptides cause a significant buckling of the membrane; however, no water pores are formed within the time scale of the simulation; (**e**) The complete 3-d view of the whole peptide-bilayer complex with lipid tails is shown, while water is not shown.

**Figure 11:**
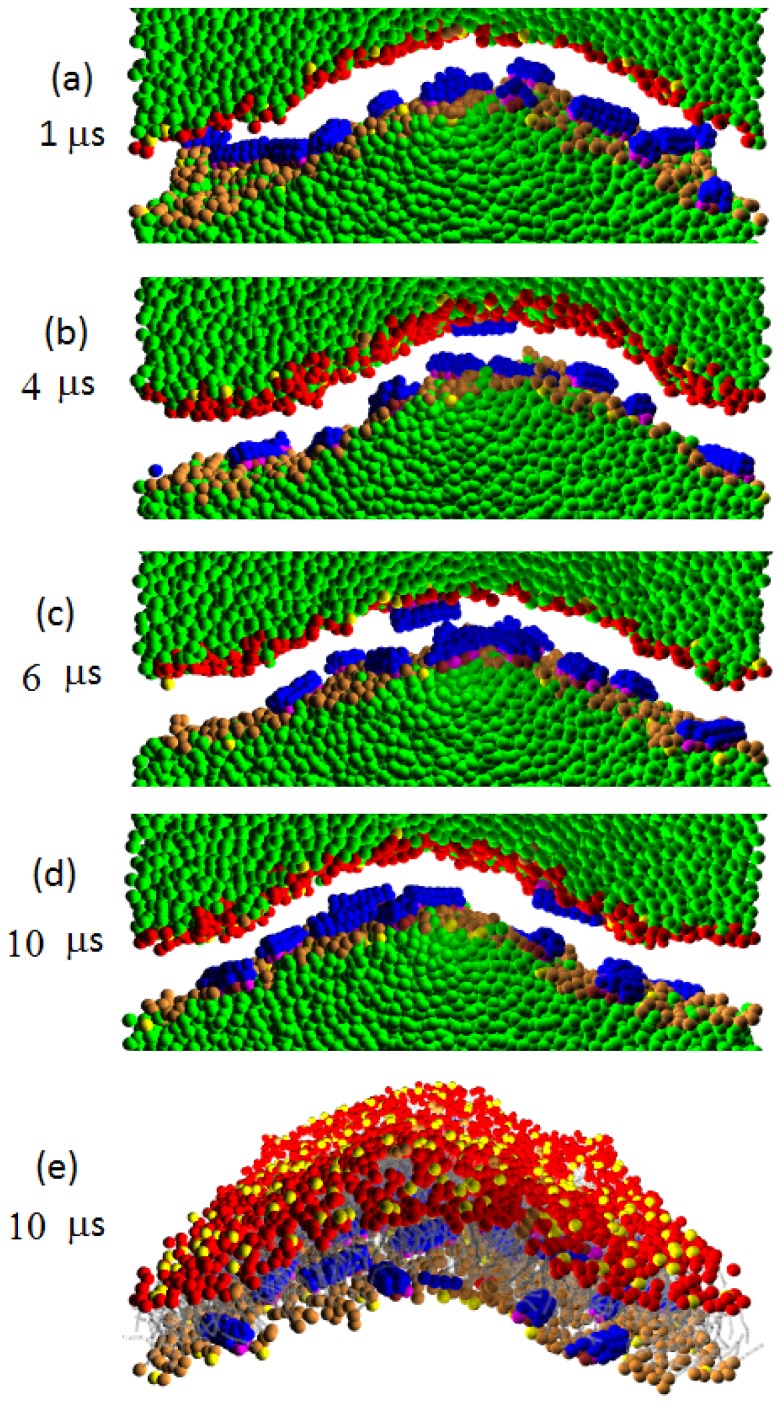
(**a**)–(**d**) Time sequence of the cross sectional images for Pep-II interacting with a W-lipid bilayer membrane simulated at *a_HW_* = 20 and *a_PhC_* = 50. In the cross sectional images, water is explicitly shown, while lipid tails are not shown for clarity. The peptides cause a significant buckling of the membrane; however, no water pores are formed within the time scale of the simulation; (**e**) The complete 3-d view of the whole peptide-bilayer complex with lipid tails is shown, while water is not shown.

**Figure 12:**
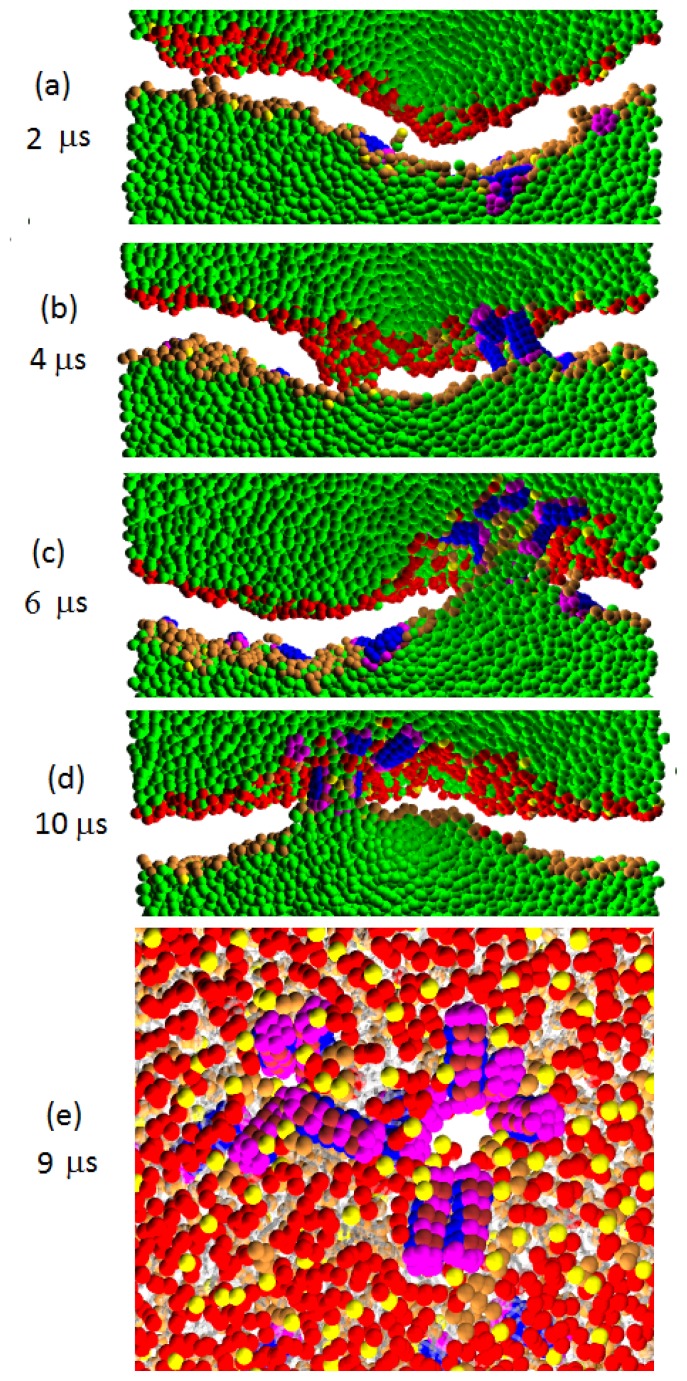
(**a**)–(**d**) Time sequence of the cross sectional images for Pep-III interacting with a W-lipid bilayer membrane simulated at *a_HW_* = 20 and *a_PhC_* = 50. In the cross sectional images, water is explicitly shown, while lipid tails are not shown for clarity. The peptides cause a significant buckling of the membrane. Note that peptides also induce the formation of a large water permeable pore; (**e**) View from above on the water pore. Water is not shown, so the pore channel appears as a “white hole”.

**Figure 13:**
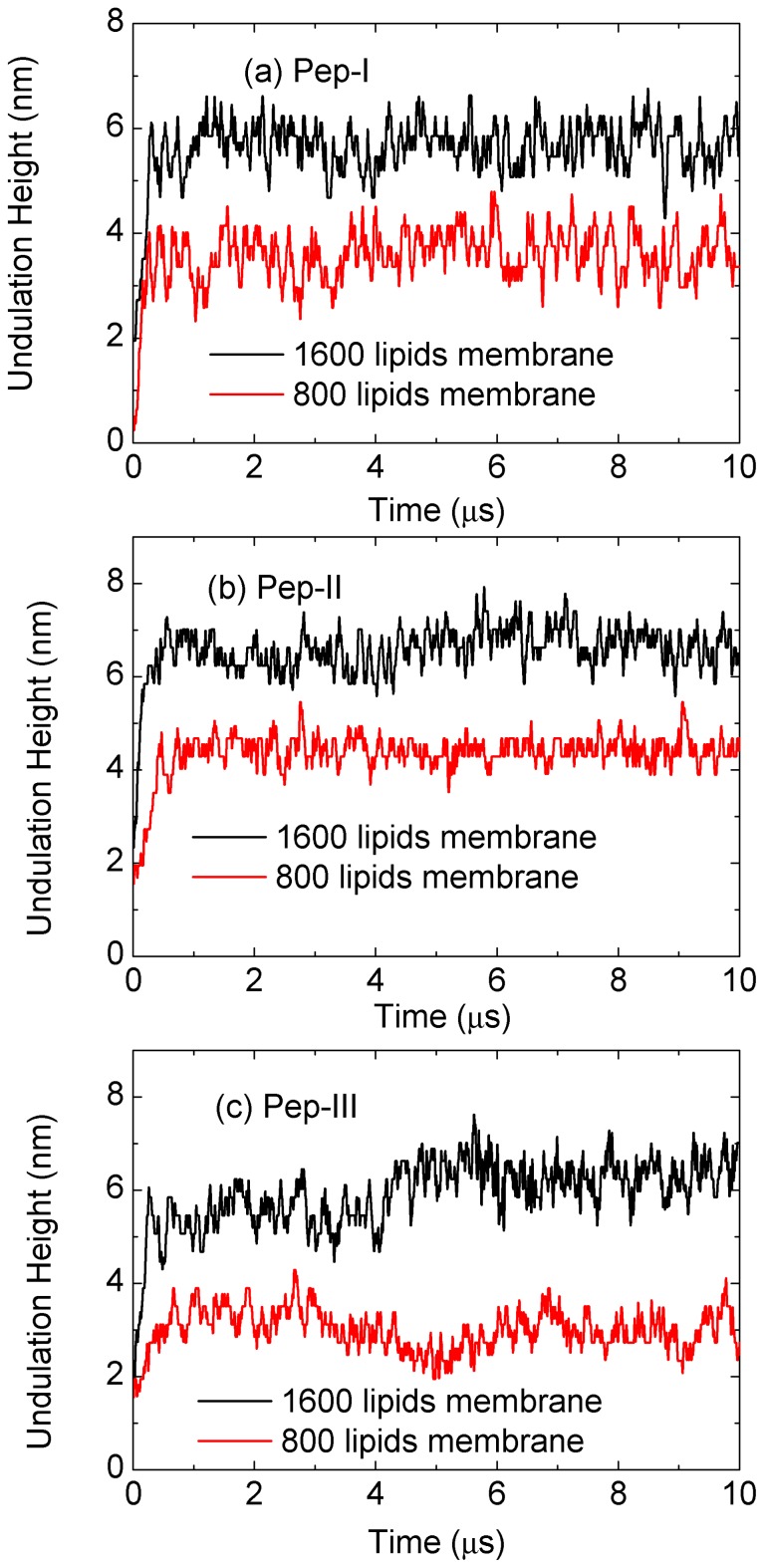
Membrane buckling magnitude in the W-lipid membrane-peptide complexes simulated at *a_HW_* = 20 and *a_PhC_* = 50 for (**a**) Pep-I; (**b**) Pep-II and (**c**) Pep-III. Black lines are for bigger membranes with 1,600 lipid molecules, while the red lines are for smaller membranes with 800 lipid molecules. The displayed buckling magnitude is defined by using the lipid heads in the upper membrane leaflet, as the height difference, *z_max_* −*z_min_*, between the highest and the lowest lipid head in the leaflet.

Figures 10, 11, 12 display the evolution of W-lipid membranes interacting with Pep-I, Pep-II and Pep-III (all with *a_PhC_* = 50), respectively. As noted above, the simulations are done in the (*NV T*) ensemble, *i.e*., with a fixed membrane base area. For the simulations in Figures 10, 11, 12, with membranes with 1,600 lipids, the membrane base area is 580 nm^2^. For comparison, we also simulated twice smaller membranes with 800 lipids, with the base area of 290 nm^2^ [see Figure 13 and the discussions in the following]. In all of the simulated cases, the peptide to lipid ratio is 2/100. Before adding peptides, all membranes were essentially flat.

A notable morphological effect seen in all these simulations is that the peptide absorption produces a substantial buckling undulation of the membranes having fixed base areas. We characterize this undulation as Euler buckling induced by the net membrane area increase caused by peptide absorption into the membrane. The mathematical signature of Euler buckling undulation is that its final (late time) magnitude is simply proportional to the linear size of the membrane base (for sufficiently large membranes). To verify this feature for the presently studied systems, in Figure 13, we plot the magnitudes of the buckling undulation for all of the three kinds of peptide interacting with membranes with the aforementioned two different sizes, with 1,600 lipids and 800 lipids. Thus, the ratio between the linear sizes of these two membranes is $\sqrt{2}\approx 1.41$. By comparing this ratio with numerical data displayed in Figure 13, we can see that the undulation amplitude (at late times) is proportional to the membrane lateral size. This is in accord with our assertion that the observed undulation is the Euler buckling. We note that, in general, the Euler buckling amplitude is proportional to the lateral length scale (wavelength) of the buckling undulation; see Refs. [21,22]. This lateral scale generally grows with time [21,22], and only at the longest times, the lateral length scale reaches the full membrane lateral size.

By Figure 13, for the membranes with 1,600 lipids, having a lateral linear size of about 24 nm, the amplitude of the Euler buckling is about 6 nm (at late times). By recalling here that the buckling amplitude is simply proportional to the lateral scale of the buckling undulation, one easily deduces that the buckling amplitude would be about 25 nm for the lateral scale of about 100 nm. These numbers are illuminating, since their orders of magnitude correspond well to the structural length scales of the membrane corrugation seen in the experiments of Fantner and co-workers [13] (see also Reference [14]). In view of this, we suggest that the experimentally observed corrugation is Euler buckling, due to the increase of the membrane area induced by the absorbed antimicrobial peptide.

We would like to stress that the Euler buckling seen here with W-lipids is of a very different nature from the membrane corrugation observed with D-lipids in Section 3. The corrugation seen in D-lipid membranes is induced by new growing lipid layers; hence, the magnitude of the resulting corrugation is typically a few nanometers in size (see Figures 2– 4, 8 and 9, at late simulation times). In the D-lipid systems, the corrugation amplitude (at late times) is primarily determined by the thickness of the new layer. In marked contrast to this, the Euler buckling reported here with W-lipids is a long scale phenomenon, with an amplitude proportional to the lateral length scale of the corrugation, as documented by Figure 13.

Because of the weaker water/lipid repulsion, W-lipids better associate with water than the D-lipids. Recall that the pores observed with D-lipids are mostly “dry”, with little water present; see Sections 3 and 4. In contrast to this, W-lipid membranes support the formation of “wet” water rich pores, as shown in Figure 12 from our simulations with Pep-III. From the figure, we see that peptides induce the formation of a large water permeable pore (in addition to the significant buckling of the membrane). Such large water permeable pores would yield an efficient cell leakage, prone to cause the cell death seen in the experiments [13,14]. Thus, our simulations with W-lipid bilayers exhibit the main elements of the experimental phenomenology [13,14]: large size membrane corrugation and the presence of water permeable pores able to cause lethal cell leakage (see Section 6 for additional discussions).

### 5.2. W-Lipid Vesicles Interacting with Peptide Molecules

**Figure 14:**
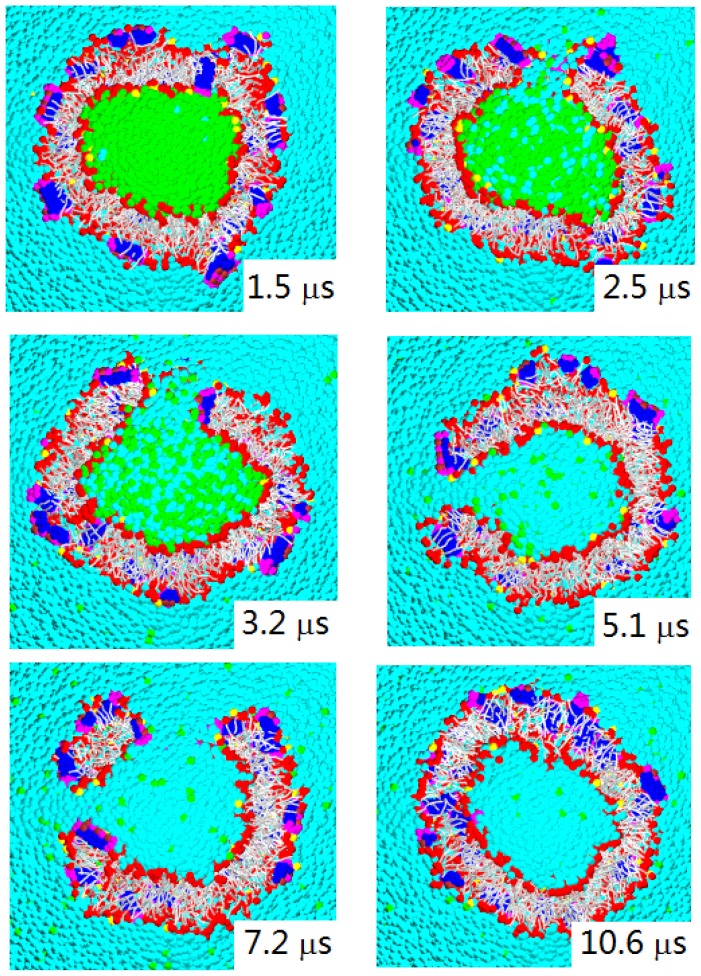
Time sequence of the cross sectional images for Pep-III interacting with a W-lipid vesicle simulated at *a_HW_* = 20 and *a_PhC_* = 50. Notable is the formation of large water permeable pores. The water pores are metastable, but long-lived with a lifetime of a few microseconds. We note that the simulated vesicle is composed of 1,200 lipids, and its initial diameter is about 14 nm.

We also simulated the interactions between W-lipid vesicles and peptide molecules, see Figure 14. The figure displays the time sequence of the cross sectional images for Pep-III interacting with W-lipid vesicle simulated at *a_HW_* = 20 and *a_PhC_* = 50. Again, we can see the formation of large water permeable pores. The pore morphology seen here corresponds well to that of the “toroidal-pore model”. In this model, the inserted peptides associate with lipid heads to form a pore. In contrast to the barrel-stave model, here, the line of the water pore is composed of both peptides and lipid head groups. Magainins, protegrins and melittins are believed to employ such a mechanism [8]. The water pores observed in our simulations are metastable, but long-lived with a lifetime of a few microseconds. That is, a pore is nucleated, and after a few microseconds, it reseals. This process is seen to cyclically repeat in the course of the simulation.

We note that the vesicle in Figure 14 is simulated with a peptide/lipid ratio of 5/100, so the pore formation is more active than in the corresponding bilayer membrane in Figure 12, simulated at a smaller peptide/lipid ratio of 2/100. Thus, there are multiple pores formed on this relatively small vesicle that are seen at 7.2 *μ*s. We saw the formation of water permeable pores only on the vesicles made of W-lipids. Such large pores are never formed on the vesicles made of D-lipids.

## 6. Discussion of W-Lipid/Peptide Complexes: Killing Mechanisms and Comparisons with D-Lipid/peptide Complexes

Our theoretical results with W-lipid/peptide complexes exhibit two major features of the recent experimental phenomenology with antimicrobial peptides [13,14]: large-scale membrane corrugation and the existence of water permeable pores that are very likely responsible for the bacterial death occurring in these *in vivo* experiments. The amplitude of the membrane corrugation seen in Reference [13] suggests that the experimental system is a W-lipid/peptide complex rather than a D-lipid/peptide complex: recall that in D-lipid/peptide complexes, the corrugation amplitude is just a few nanometers, which is too small to explain the experimental data on the membrane roughness reported in Reference [13] (see also the Supplemental Information of the cited paper). On the other hand, as discussed in Section 5.1, the Euler buckling observed in our simulations of W-lipid/peptide complexes is a long length scale phenomenon that can explain well the magnitude of the experimentally observed membrane roughness. In addition to the buckling, the W-lipid/peptide complexes exhibit large water permeable pores seen in our simulations with W-lipid bilayers (Section 5.1) and W-lipid vesicles (Section 5.2) interacting with peptide molecules. The presence of such pores may well lead to the bacterial death seen in Reference [13]. It did not escape our attention that our W-lipid/peptide complexes behavior corresponds well to widely adopted picture of antimicrobial peptide actions, which associates peptide lethality to water permeable pores and cell leakage.

Thus, our study of W-lipid/peptide complexes vindicates (on a more microscopic level) the frequently adopted qualitative picture of these phenomena. In addition, our study has also introduced the new concept of D-lipid/peptide complexes. These complexes exhibit the formation of cores (new layers), which may also affect the cell functions, see the discussions in Section 4. Let us discuss the physics behind the observed differences between “dry” D-lipids and “wet” W-lipids: The microscopic difference between them is in the strengths of the repulsive interaction between the lipid heads and water (the *a_HW_* parameter) and the repulsive interaction between the lipid heads (the *a_HH_* parameter, which is conveniently set to be = *a_WW_*). For D-lipids, *a_HW_> a_HH_*, and this condition gives rise to an affinity of D-lipid heads to better associate with each other (HH affinity) and separate from water, because of the stronger HW repulsion (relative to the HH repulsion). This tendency favors the formation of the “dry” lipid head cores (new layers) seen in D-lipid/peptide complexes in Section 3. Indeed, in most of the cases studied in Section 3, the observed cores are dry with little water present. Likewise, the pores seen in D-lipid complexes are also mostly dry wormhole type pores. On the other side, for W-lipids, *a_HW_< a_HH_*, and this condition gives rise to the affinity of W-lipid heads to better associate with water, because of the weaker HW repulsion (relative to the HH repulsion). Due to this, the peptide nucleated pores in W-lipid membranes are water-rich and water permeable. At the same time, the condition, *a_HW_< a_HH_*, suppresses the formation of the cores (new lipid head layers) in W-lipid/peptide complexes, because of the (relatively) stronger HH repulsion. This condition favors intermixing of lipid heads with water, *i.e*., the HW association over the HH association. Hence, no new lipid head layers are formed in W-lipid/peptide complexes. Rather, the main peptide induced effects in W-lipid/peptide complexes are the formation of water-rich permeable pores and Euler buckling induced by peptide absorption into membranes.

## 7. Summary

In summary, we have explored the actions of peripheral and transmembrane antimicrobial peptides on the lipid bilayer membrane by using the coarse-grained Dissipative Particle Dynamics simulations. Our study is aimed to reach the bigger picture of these phenomena. In view of this, we have elucidated peptide/lipid membrane complexes by considering peptides with various structure, hydrophobicity and peptide/lipid interaction strength. The role of lipid/water interaction is also elucidated. We have discussed a rich variety of membrane morphological changes induced by peptides, such as pore formation, membrane corrugation and Euler buckling. We find that lipid/peptide complexes can be sorted into two major categories: (i) D-lipid/peptide complexes, exhibiting formation of new lipid layers and (ii) W-lipid/peptide complexes exhibiting Euler buckling (and no new layer formation).

Comparison between our theory and recent experimental phenomenology [13,14] suggests that these experimental systems are W-lipid/peptide complexes. We attribute the experimentally observed bacterial death [13] to water permeable toroidal pores seen in our simulations of W-lipid/peptide complexes, which, much like the experimental systems [13], exhibit large-scale membrane corrugation elucidated here as Euler buckling. Our study has also introduced the new concept of D-lipid/peptide complexes exhibiting formation of new lipid layers. This interesting theoretical prediction will inspire future experimental and theoretical work in the broad area of biological membrane physics. There are so many different membrane systems, including the membranes of not only bacteria, but also of fungi or even mammals, and it is interesting to know how antimicrobial peptides interact with all of them. One can ask significant questions, such as how the antimicrobial peptides are actually non-lethal to some organisms, and many other such questions related to diverse membrane systems. We tried to cover diverse membrane systems as much as it is possible within our theoretical approach. It is thus hoped that our study will help to paint a bigger and more general physical picture of the phenomena involving interactions between diverse biological membranes and peptide molecules. Finally, we would like to note that peptides can also induce significant effects in multi-lamellar membrane phases, see, e.g., Reference [23]. An interesting extension of the present theory is to address complexes of peptide with a wide variety of multi-membrane phases [24–26] as well as with tethered (polymerized) membranes [27].

## Figures and Tables

**Table 1:** Force parameters *a_ij_* (in units of *k_B_T=r*_0_). H, C, W, Ph and Pc stand for the head bead of lipid, tail bead of lipid, water bead, hydrophilic bead of the peptide and hydrophobic bead of the peptide, respectively. For D-lipids, *a_HW_* = 35, while for W-lipids, *a_HW_* = 20.

*a_ij_*	H	C	W	Ph	Pc
H	25	50	35/20	25	50
C	50	25	75	25/35/50	25
W	35/20	75	25	35	120/500
Ph	25	25/35/50	35	25	25
Pc	50	25	120/500	25	25

**Table 2:** Statistics of peptide translocation across D-lipid membranes. The minimum, maximum and average number of translocated peptides, as well as the number of events of hydrophilic discoidal core formation for the three peptide models simulated with *a_PhC_* = 25, 35 and 50 are listed. Data are obtained from five independent samples for each case simulated up to 10 *μ*s.

	Pep-I (25/35/50)	Pep-II (25/35/50)	Pep-III (25/35/50)
$N_{Min}^{tran}$	0/9/0	0/0/0	0/1/0
$N_{Max}^{tran}$	7/10/2	2/10/7	2/6/1
$N_{ave}^{tran}$	5/10/1	1/6/3	1/4/1
*N_Core_*	2/0/4	5/2/1	0/3/5
